# Embedding voices of under-served communities in Health Research Priority Setting in Thailand

**DOI:** 10.12688/wellcomeopenres.22940.1

**Published:** 2024-10-29

**Authors:** Napat Khirikoekkong, Supa-at Asarath, Anne Osterrieder, Khin Maung Lwin, Phaik Yeong Cheah

**Affiliations:** 1 Mahidol Oxford Tropical Medicine Research Unit, Mahidol University, Bangkok, Bangkok, 10400, Thailand; 2 Nuffield Department of Medicine, Centre for Tropical Medicine and Global Health, University of Oxford, Oxford, England, OX3 7LF, UK; 3 Shoklo Malaria Research Unit, Mahidol University, Mae Ramat, 63140, Thailand

**Keywords:** Health research priority setting, ethics, community advisory board, Thailand

## Abstract

Health research is needed to develop new treatments, diagnostic methods and prevention strategies for many diseases, but resources are limited particularly in low-and middle-income countries (LMICs). Our proposed project involves in-depth engagement with community advisory boards and existing community groups in healthcare research priority setting in Thailand. We will use Wellcome’s “Responsive Dialogues” engagement approach, focusing on “underserved” groups, as their voices are seldom heard in healthcare research priority setting. We estimate that we will engage with 6–8 community groups involving 48–80 participants. Our objectives are firstly, to contribute to the health research agenda setting in Thailand for government, non-governmental actors, academia, and others who conduct health research in the next 5 to 10 years. Secondly, we aim to identify ethical and practical issues related to healthcare research priority setting, specifically in relation to involving under-served communities.

## Plain language summary

Health research is essential for developing new treatments, diagnostic methods and prevention strategies for many diseases, but resources are limited limited particularly in low-and middle-income countries (LMICs). Our proposed project aims to involve community advisory boards and local community groups in deciding health research priorities, using a series of interactive dialogues. We will focus on under-served groups, whose voices are rarely heard in these decisions, and plan to engage with 6-8 community groups, involving 48-80 participants. Our goals are to help inform health research priorities in Thailand for the next 5 to 10 years, guiding government, non-governmental organizations, academics, and others who conduct health research, and to identify ethical and practical issues related to involving community advisory boards and local groups in this process.

## Introduction

Health research is needed to develop new treatments, diagnostic methods, and prevention strategies for many diseases, but resources are limited particularly in low-and mid-income countries (LMICs). This necessitates difficult decisions to allocate these scarce resources, making the prioritization of health research essential ^1^. Healthcare research priority setting should promote health equity as a matter of justice ^2^. This process requires the involvement of a diverse range of stakeholders, including research producers, research users, and research beneficiaries ^3^. However, formal priority setting is seldom conducted, and input from research beneficiaries, such as patients, carers, and communities, is not frequently solicited. The involvement of under-served communities in healthcare research priority setting is even more uncommon. Engaging these communities can amplify their voices and concerns in research priority setting, ensuring that research topics and questions are more explicitly focused on improving their access to and affordability of healthcare and services. It is hoped that getting input from these groups means research priority setting will be more ‘comprehensive’, defined as ‘presence required to achieve complete or, at least, a wide representation of the community or society’s population’ ^4^.

The engagement of underserved groups and communities has been proposed as a core component of equity-oriented health research priority setting ^3^, but how this should be done is not clearly detailed. There are challenges associated with engaging these communities, such as low literacy, low health and science literacy, power dynamics, and limited resources for priority setting.

For the purpose of this project, we adopt the UK National Institutes of Health Research (NIHR) definition of under-served communities as “a group that is less well represented in research than would be desirable from population prevalence and healthcare”. Their perspectives and needs are rarely included in the processes that shape research agendas. This exclusion extends to national priority-setting exercises, where the voices of these populations are often overlooked.

Our proposed project involves in-depth engagement with under-served communities in healthcare research priority setting. It will be situated in Thailand, where our administrative and research hub is located. Thailand is an upper-middle income country with a population of approximately 71.9 million people ^5, 6^. We focus our efforts on engagement with community advisory boards and existing community groups, such as the Tak Province Community Ethics Advisory Board ^7– 9^. We chose these groups because they are existing patient and community interest groups who meet regularly, are familiar with each other, familiar with health-related research and are used to providing feedback to researchers. More importantly, some of these groups comprise under-served communities such as migrants, ethnic minorities, hill tribe communities, and young people. The members of these groups are not necessarily representative of the entire community, but they are often typical of it.

We will engage with young people, as their voices are rarely heard in research. The United Nations Convention on the Rights of the Child (CRC) encourages the participation of young people in evidence generation and policymaking ^10^. It outlines the obligation of states to create an environment that allows the views of children and adolescents to be heard on practices and policies that affect them. We acknowledge that various governmental and non-governmental actors, as well as academic institutions conducting health research in Thailand, have engaged in priority-setting exercises. However, our project will focus on under-served groups to generate a supplemental list of priorities that reflect what is most important to these populations in Thailand.

We envisage that results of this project will be used in two different ways. Firstly, they are intended to contribute to the health research agenda setting by identifying health needs that are not obvious from existing data sources or other priority setting exercises such as those conducted by government agencies. This initiative aligns with Thailand’s Country Cooperation Strategy (CCS) 2022–2026, which emphasizes improving health outcomes for all population groups, particularly “vulnerable” communities, including migrants who often face multi-layered barriers to accessing healthcare and services ^11^. The World Bank, WHO, and Thailand's National Economic and Social Development Plan all support inclusive approaches that integrate the health needs of all populations, especially those who are most vulnerable and often neglected in traditional research priority settings. Secondly, our experience of engaging with these groups will serve as a case study of how such engagement can be conducted and what challenges it may bring.

## Objectives

Primary Objective: To gather input from under-served communities to inform and supplement existing health research priorities in Thailand for the next 5 to 10 years.

Secondary Objective: To identify ethical and practical issues related to healthcare research priority setting, specifically in relation to involving under-served communities.

## Methods

### Approach

This is an engagement project. We will employ a modified version of Wellcome’s Responsive Dialogues approach which emphasizes open and inclusive conversations. This approach has three phases: groundwork, “community conversations”, and feedback to participants ^12^. Groundwork involves conducting literature review and talking to healthcare staff about the health needs and problems that the communities face. Community conversations are dialogues that we will be having with these communities. These dialogues will be facilitated by members of the project team who have experience and training in public engagement, facilitation and are specifically trained in using Wellcome Trust’s “Responsive Dialogues” framework ^13, 14^.

Facilitators are experienced in engaging with under-reached groups. They are fluent in English, Thai, and Karen, and they will be supported by local facilitators familiar with the participants’ culture and languages. This ensures that participants can freely express their views in their native tongue, making them feel more comfortable and open during discussions. In keeping with the ethos of Responsive Dialogues, a small number of researchers may be invited to each session to engage directly with participants. Sessions will be held in the usual locations for community meetings and will be audio-recorded to ensure accurate note-taking and capture key insights. Transcribed files will be securely stored with password protection and accessible only to the project team.

Facilitators will use a facilitators’ guide (see extended data) specifically designed to foster conversations and dialogues. This guide will help keep discussions focused, provide example probing questions, and offer reminders for facilitators throughout the session. The guides will be tailored to each group to ensure they are appropriate, avoiding terminology or references that could cause misunderstandings or raise unrealistic expectations. For example, in most of the conversations, we will use the term “a conversation/dialogue on health and health research topics” rather than “priority setting exercise”. After each session, the project team will hold reflection meetings to adjust our approach and make improvements for future conversation events.

Each session will last between 2 to 3 hours. We anticipate that 2 to 3 sessions will be required for each community or advisory group to cover all necessary topics and ensure thorough engagement.

In addition to the community conversations, participatory visual methods may be used to supplement the conversations. This approach will help to visualize and contextualize the issues being discussed, providing a richer and more nuanced understanding of the participants' perspectives

After we conduct all the community conversations, we will organise feedback sessions to the same communities.

### Participants

The focus of this project is on under-served communities, such as hill tribe members, migrants, and others whose voices are seldom heard in the space of health research. We also make an effort to include young people 18 to 24 years old. Specific participant groups include:

- Existing community advisory boards in Thailand e.g. Tak Province Community Ethics Advisory Board which consists of adult migrants from Myanmar living on the Thai-Myanmar border zones ^7, 8^, the Chiang Rai Hilltribe Advisory Board which consists of hill tribe communities living in the Chiangrai province
- Existing youth groups e.g. Tak Province Young Persons’ Advisory Group which consists of migrants between 18 and 24 years old
- Other existing community or patient groups or “patient and public involvement” (PPI) groups (e.g. melioidosis survivors’ group in Ubon Ratchathani, tuberculosis patients and families group in the Tak Province)

### Sample size

We estimate that we will engage with 6–8 community groups involving 48–80 participants.

### Data analysis and output

The data collected from these sessions will in terms of meeting minutes and team reflections. They will be analysed using thematic analysis. This method will allow us to identify, analyse, and report patterns (themes) within the data, ensuring a comprehensive understanding of the key issues and insights generated from the conversations.

The output will be a report to inform health researchers in government, non-governmental sectors, academia, and others who conduct health research in Thailand.

### Timeline

Preparation and piloting: August and September 2024

Duration of project: September 2024 to August 2025

## Ethical considerations

Participants are existing members of these community groups. They will be invited to participate in these priority-setting sessions, with the choice to attend or not. It will be made clear that participation is entirely voluntary, and participants may withdraw from the sessions at any time without any negative consequences. Since the facilitators are engagement practitioners from an academic institution, and not healthcare providers or government officials, we hope this will minimize any perceived power differences between the facilitators and participants.

Those who choose to attend will be asked to register for the conversation event. In the locations where we plan to hold these conversations, registering for an event is commonly understood as active consent to participate, which is a standard practice in the region. We will securely store all written registration forms in compliance with the Thailand Data Protection Act of 2019.

The project team and the facilitators of these groups will take the time to explain the purpose of the project as thoroughly as possible to all participants. Throughout all interactions, the project team will remain mindful of potential power dynamics influenced by age, social hierarchy, and gender, ensuring that all voices are heard and respected. We are cognizant that not all health needs can be addressed so we will be careful not to raise unrealistic expectations among participants.

All events will undergo a risk assessment in accordance with our safeguarding policy before they are held. This assessment will identify any potential risks to participants, especially vulnerable groups, and outline measures to minimize, mitigate, or address these risks. Additionally, participants will be compensated for their time, recognizing their contributions and encouraging meaningful engagement.

## Limitations

We confine our project to existing community advisory boards and community groups. It is not possible to identify all under-served communities.

## Ethics and consent

This is an engagement project, not a research study. We have obtained a waiver for ethics review from our ethics committee, the Oxford Tropical Research Ethics Committee (OxTREC).

## Data Availability

No data are associated with this article. Zenodo: Embedding voices of under-served communities in Health Research Priority Setting in Thailand: Facilitators' Guide. https://doi.org/10.5281/zenodo.13751509 ^15^ This project contains the following extended data: Facilitators' Guide_Priority Setting Engagement Project.pdf Data are available under the terms of the Creative Commons Attribution 4.0 International license (CC-BY 4.0).
