# Supplementary material for: Embedding voices of under-served communities in Health Research Priority Setting in Thailand
Source: Wellcome Open Res. 2025 Feb 18;9:627. Originally published 2024 Oct 29. [Version 2] doi: 10.12688/wellcomeopenres.22940.2 (PMC11883205; doi:10.12688/wellcomeopenres.22940.2)
Supplement: Peer review history

# Reviewer report: Reviewer response for version 2

**DOI:** 10.21956/wellcomeopenres.26258.r119019

**Published:** 2025-03-06

**Authors:** Sarah Saleem

**Affiliations:** 1 Aga Khan University, Karachi, Pakistan

This is a proposal to priorities health research agenda by involving underserved communities in pre identified areas in Thailand . The authors propose to engage community advisory boards, existing community groups and young people from underserved communities. The purpose of the activity is to give voice to underserved communities for setting health research agenda for Thailand government and non governmental actors and academia interested in conducting health research. For this the authors will use two main methods 1) use a modified version of Wellcome’s “Responsive Dialogues” approach which emphasises open and inclusive conversations having three phases; the groundwork, community conversations, and feedback to participants. the second method is adding  Participatory Visual Methods (PVM)  to supplement the conversations. The trained persons will conduct the session. The suggested names for the groups are mentioned in the proposal. The limitations are clearly given.

Is the study design appropriate for the research question?

Yes

Is the rationale for, and objectives of, the study clearly described?

Yes

Are sufficient details of the methods provided to allow replication by others?

Yes

Are the datasets clearly presented in a useable and accessible format?

Not applicable

Reviewer Expertise:

Community Health specially RH, epidemiology , maternal and newborn health

I confirm that I have read this submission and believe that I have an appropriate level of expertise to confirm that it is of an acceptable scientific standard.

# Reviewer report: Reviewer response for version 2

**DOI:** 10.21956/wellcomeopenres.26258.r118887

**Published:** 2025-03-05

**Authors:** Kate Fryer

**Affiliations:** 1 University of Sheffield, Sheffield, UK

I have reviewed the revisions and feel happy to alter by status to Approved, with no reservations.

Is the study design appropriate for the research question?

Partly

Is the rationale for, and objectives of, the study clearly described?

Yes

Are sufficient details of the methods provided to allow replication by others?

No

Are the datasets clearly presented in a useable and accessible format?

Not applicable

Reviewer Expertise:

Co-production methodologist with underserved communities.

I confirm that I have read this submission and believe that I have an appropriate level of expertise to confirm that it is of an acceptable scientific standard.

# Reviewer report: Reviewer response for version 2

**DOI:** 10.21956/wellcomeopenres.26258.r118886

**Published:** 2025-02-25

**Authors:** Joseph Millum

**Affiliations:** 1 University of St Andrews, Scotland, UK

The authors have provided a thoughtful and thorough response to the reviewer comments. I have no further comments and wish them all the best with the project.

Is the study design appropriate for the research question?

Partly

Is the rationale for, and objectives of, the study clearly described?

Partly

Are sufficient details of the methods provided to allow replication by others?

Partly

Are the datasets clearly presented in a useable and accessible format?

Not applicable

Reviewer Expertise:

Ethics, priority setting.

I confirm that I have read this submission and believe that I have an appropriate level of expertise to confirm that it is of an acceptable scientific standard.

# Reviewer report: Reviewer response for version 1

**DOI:** 10.21956/wellcomeopenres.25260.r110205

**Published:** 2024-11-26

**Authors:** Joseph Millum

**Affiliations:** 1 University of St Andrews, Scotland, UK

This is an exciting and potentially very valuable project which aims to engage underserved communities in Thailand and feed information from this engagement into national research priority setting.  

**Major comments:**

 1. My main concern is with the non-representative nature of the communities whose needs will be elicited. If the results are used to inform national priority setting, but they are not representative of the needs of underserved communities overall in Thailand, then the priorities will be skewed. For example, if patient groups that represent some diseases but not others are consulted, then research on those diseases is more likely to get prioritized as a result of the project. The authors should explain how this sort of problem can be avoided (without trying to engage a representative set of community groups, which would be impractical).

 2. Related, on p.3, the authors write: “The members of these groups are not necessarily representative of the entire community, but they are often typical of it.”

      The meaning of “typical” should be explained. If they are not representative, then is there still a sense in which the members can speak on behalf of the communities?

 3. In some places, it seems as though the engagement is intended to find out about health needs, in other places it seems as though participants will also be asked about their health research priorities. The scope of the topics and how the information gathered will feed into research priority setting could be specified more clearly.

 4. Will there be an output corresponding to the second objective?  

**Minor comments:**

 1. p.3. “‘comprehensive’, defined as ‘presence required to achieve complete or, at least, a wide representation of the community or society’s population.’”    

 I didn’t see how this was a definition of comprehensive. Clarify.

 2. p.3. “definition of under-served communities as” Should be “community”

Is the study design appropriate for the research question?

Partly

Is the rationale for, and objectives of, the study clearly described?

Partly

Are sufficient details of the methods provided to allow replication by others?

Partly

Are the datasets clearly presented in a useable and accessible format?

Not applicable

Reviewer Expertise:

Ethics, priority setting.

I confirm that I have read this submission and believe that I have an appropriate level of expertise to confirm that it is of an acceptable scientific standard, however I have significant reservations, as outlined above.

# Response

**Published:** 2025-02-11

**Authors:** Phaik Yeong Cheah

**Affiliations:** Mahidol Oxford Tropical Medicine Research Unit, Thailand

Thank you for your valuable comments.  

 This is an exciting and potentially very valuable project which aims to engage underserved communities in Thailand and feed information from this engagement into national research priority setting.    

**Response: **Thank you.

**Major comments:**

 1. My main concern is with the non-representative nature of the communities whose needs will be elicited. If the results are used to inform national priority setting, but they are not representative of the needs of underserved communities overall in Thailand, then the priorities will be skewed. For example, if patient groups that represent some diseases but not others are consulted, then research on those diseases is more likely to get prioritized as a result of the project. The authors should explain how this sort of problem can be avoided (without trying to engage a representative set of community groups, which would be impractical).

**Response:** We acknowledge that the communities involved are not representative. Our findings are intended to supplement other priority-setting exercises, such as national priority-setting initiatives, which may not include any under-served communities. When reporting, we will ensure that we provide detailed explanations of who we engaged with, and clearly outline the limitations, including the possibility that the priorities of these communities may be influenced by their unique experiences of health issues and circumstances. In light of this comment, we have decided not to engage with groups with specific diseases e.g. meliodosis survivors, tuberculosis patients. We have now amended the section on ‘participants’ to reflect this change.   We have also expanded the limitations section to include this. See second paragraph under “Strengths and Limitations”.

 2. Related, on p.3, the authors write: “The members of these groups are not necessarily representative of the entire community, but they are often typical of it.”The meaning of “typical” should be explained. If they are not representative, then is there still a sense in which the members can speak on behalf of the communities?  

**Response: **We have revised the sentence to “The members of these groups are not necessarily representative of the entire community, but they are often knowledgable of the community”.   Participants are not expected to speak on behalf of the community. Through this process, we aim to ensure a range of voices is heard and considered in the research priorities set by those in positions of authority. We will make this clear to the participants. We have updated the “Strenghts and Limitations” section.

 3. In some places, it seems as though the engagement is intended to find out about health needs, in other places it seems as though participants will also be asked about their health research priorities. The scope of the topics and how the information gathered will feed into research priority setting could be specified more clearly.  

**Response:** Some of the communities would not have heard about health research so the discussion will have to start with health needs. We have now made this clearer. See paragraph 3 under “Methods”.

 4. Will there be an output corresponding to the second objective?  

**Response: **Yes. We will publish an academic paper. We have expanded the section on ‘Data analysis and output’.

**Minor comments:**

 1. p.3. “‘comprehensive’, defined as ‘presence required to achieve complete or, at least, a wide representation of the community or society’s population.’”    

 I didn’t see how this was a definition of comprehensive. Clarify.

**Response: **this is directly lifted from Pratt’s paper. We have expanded this to improve clarity. Ref: Pratt B. Inclusion of marginalized groups and communities in global health research priority-setting. J Empir Res Hum Res Ethics. 2019;14(2):169–181. 30866721 10.1177/1556264619833858

 2. p.3. “definition of under-served communities as” Should be “community”  

**Response:** Corrected.

# Reviewer report: Reviewer response for version 1

**DOI:** 10.21956/wellcomeopenres.25260.r110197

**Published:** 2024-11-11

**Authors:** Kate Fryer

**Affiliations:** 1 University of Sheffield, Sheffield, UK

**Abstract**

 Provides a clear overview of the study.

**Plain language summary**

 The phrase 'interactive dialogues' is not a lay term.

**Introduction**

 The second paragraph only details challenges felt to be posed by communities - this would be better balanced by acknowledging the academic and clinical institutions and processes which perpetuate lack of engagement.

 Third paragraph should read 'National Institute for Health and Care Research'. Also, you could use the James Lind as an example for the final sentence of this paragraph.

 Paragraph four - I think it is important to acknowledge the limitations of using pre-existing groups - you are consulting people who are already engaged.

**Objectives**

 Very clear

**Methods**

 First sentence under Data analysis and output doesn't make sense.

 Methods are clear in terms of how the groups will be identified and who will run sessions. The process of priority setting isn't clear. How will community members be supported to reach decisions on what their priorities are? What is the process of moving from discussion to decision? It reads more like a qualitative focus group about what communities think is important. 

 It would also be good to have more detail about how the findings of the study will be shared with those currently in control of the research agenda, which will help the reader to understand its potential for impact.

**Ethical considerations**

 I think you need to acknowledge potential ethical issues with using local facilitators.

 I'm also a bit confused as to why ethics is not necessary, when the sessions are being recorded an analysed as data.

Is the study design appropriate for the research question?

Partly

Is the rationale for, and objectives of, the study clearly described?

Yes

Are sufficient details of the methods provided to allow replication by others?

No

Are the datasets clearly presented in a useable and accessible format?

Not applicable

Reviewer Expertise:

Co-production methodologist with underserved communities.

I confirm that I have read this submission and believe that I have an appropriate level of expertise to confirm that it is of an acceptable scientific standard, however I have significant reservations, as outlined above.

# Response

**Published:** 2025-02-11

**Authors:** Phaik Yeong Cheah

**Affiliations:** Mahidol Oxford Tropical Medicine Research Unit, Thailand

We thank the reviewers for the valuable comments.   Here is a point by point response to reviewer comments.  

**Abstract**

 Provides a clear overview of the study.  

**Response: **Thank you.

**Plain language summary**

 The phrase 'interactive dialogues' is not a lay term.  

**Response:** We have channged ‘interactive dialogues’ to ‘conversations’

**Introduction**

 The second paragraph only details challenges felt to be posed by communities - this would be better balanced by acknowledging the academic and clinical institutions and processes which perpetuate lack of engagement.  

**Response:** We have added a sentence with examples of institutional barriers and relevant references.

 Third paragraph should read 'National Institute for Health and Care Research'. Also, you could use the James Lind as an example for the final sentence of this paragraph.  

**Response:** Done. We have included a link to a website and a reference containing lists/comparisons of national priority-setting frameworks. This list includes the James Lind Framework.

 Paragraph four - I think it is important to acknowledge the limitations of using pre-existing groups - you are consulting people who are already engaged.  

**Response: **We have updated the limitations section to include this. However, we feel that this also poses a strength for the project. We have added text ‘Strengths and limitations’.

**Objectives**

 Very clear  

**Response:** Thank you.

**Methods**

 First sentence under Data analysis and output doesn't make sense.  

**Response: **We have revised this sentence.

 Methods are clear in terms of how the groups will be identified and who will run sessions. The process of priority setting isn't clear. How will community members be supported to reach decisions on what their priorities are? What is the process of moving from discussion to decision? It reads more like a qualitative focus group about what communities think is important. 

**Response:** The process of transitioning from discussing health needs to identifying health research priorities will be guided by discussions and consensus, facilitated by our experienced facilitators. We anticipate that each community conversation will yield 3–5 broad categories of research priorities.

 It would also be good to have more detail about how the findings of the study will be shared with those currently in control of the research agenda, which will help the reader to understand its potential for impact.  

**Response: **Findings will be compiled into a report made accesssbile to those in control of the research agenda. We have expanded the section on ‘Data analysis and output’.

**Ethical considerations**

 I think you need to acknowledge potential ethical issues with using local facilitators. 

**Response:** Thank you. We have added the following sentences: We also acknowledge potential ethical issues associated with using local facilitators. The facilitators may know the participants or their families personally, which could influence what participants choose to say or withhold.

 I'm also a bit confused as to why ethics is not necessary, when the sessions are being recorded an analysed as data.

**Response: **Sessions are only recorded for minute taking which is common in most meetings in Thailand (e.g. office meetings, community meetings). We will comply with the Thailand Data Protection Act of 2019. We have consulted with our ethics committee and this was the advice they gave us. In the light of the reviewer’s comment, we will discuss this again with them.
